# Dynamic Beam Control-Based Neighbor Discovery Protocol for Underwater Acoustic Networks with Multi-Parallel Transceiver

**DOI:** 10.3390/s26061855

**Published:** 2026-03-15

**Authors:** Jianjun Zhang, Lin Zhou, Haijun Wang, Zhiyong Zeng, Qing Hu

**Affiliations:** 1 School of Ocean Engineering and Technology, Sun Yat-sen University & Southern Marine Science and Engineering Guangdong Laboratory (Zhuhai), Zhuhai 519000, China; zhangjj233@mail2.sysu.edu.cn (J.Z.); zhoulin67@mail2.sysu.edu.cn (L.Z.); wanghj59@mail2.sysu.edu.cn (H.W.); zengzhy28@mail2.sysu.edu.cn (Z.Z.); 2 Zhuhai Research Center of Hanjiang National Laboratory, Zhuhai 519000, China; 3 Guangdong Provincial Key Laboratory of Information Technology for Deep Water Acoustics, Zhuhai 519000, China

**Keywords:** underwater acoustic networks, neighbor discovery, direction of arrival, directional transceiver, multi-beam parallel transmission

## Abstract

Neighbor discovery in underwater acoustic networks (UANs) faces challenges such as high propagation delay and limited spectrum resources. This study proposes a dynamic beam control-based multi-parallel transceiver neighbor discovery protocol (DBCB), which improves node discovery efficiency by dynamically matching transmission beams and optimizing spatiotemporal frequency resource allocation. During node initialization, the master node broadcasts omnidirectionally to quickly capture coarse-grained neighbor parameters. After obtaining these parameters, the master node dynamically allocates orthogonal frequency bands for directional multi-beam validation and optimizes beam alignment, resource allocation, and topology stability through real-time feedback. The protocol adaptively optimizes transmission power and continues the discovery task, while nodes that remain undiscovered for extended periods automatically adjust their receiving gain. The adaptive power control mechanism adjusts the transmission power based on node distance and azimuth, enabling the protocol to maintain low power consumption and enhance interference resilience. Simulation results show that the DBCB protocol outperforms similar neighbor discovery protocols based on directional transmission-reception (DTR) and random two-way (RTW) mechanisms, with improvements of 7.84% and 28.17% in average discovery rate, and reductions of 28.13% and 59.06% in average discovery delay, respectively. The anechoic tank experiment demonstrates that multi-beam parallel transmission effectively improves underwater node discovery efficiency, with simulation results aligning with experimental data, confirming the stability and high efficiency of the system.

## 1. Introduction

Underwater Acoustic Networks (UANs) are indispensable for a wide range of marine applications, including environmental monitoring, marine exploration, and Autonomous Underwater Vehicle (AUV) coordination [1]. Neighbor node discovery is a prerequisite for efficient network formation. Due to the unique nature of underwater signal propagation, neighbor discovery must overcome challenges such as signal attenuation, noise interference, and multipath effects [2,3,4]. These factors significantly increase the complexity of fundamental network functions such as neighbor discovery. Rapid and accurate neighbor discovery is crucial for topology establishment, routing initialization, and collaborative sensing [5]. As a key component of underwater acoustic communication, neighbor node discovery and localization are responsible for coordinating node access to shared communication resources [6]. Their performance directly determines the overall network efficiency, throughput, latency, and reliability [7,8]. Traditional neighbor discovery mechanisms in UANs often face issues of long delays and low success rates due to the specific limitations of acoustic channels [9]. Some time-based MAC protocols only address collisions by slot allocation, neglecting the optimization of spatial and frequency dimensions, leading to resource waste in space and frequency [10]. Similarly, some space- or frequency-based protocols also exhibit similar limitations, including inefficient resource utilization, limited throughput, and high energy consumption, making it difficult to effectively cope with the complex and dynamic underwater communication environment [11,12].

Traditional underwater acoustic networks typically use omnidirectional transducers for communication. In omnidirectional transducer-based systems, communication nodes share the same channel, which severely limits network performance and makes it difficult to fully realize the potential of underwater networks [13]. Nodes transmit the same power of acoustic signals in all directions simultaneously, causing substantial interference with neighboring nodes. This leads to packet collisions, reduces network throughput, and hinders covert communication [14,15]. Directional communication, using multi-directional sensors and directional communication techniques, allows networks to reduce interference between neighboring nodes, improving transmission efficiency and reducing energy consumption [16]. Liu et al. proposed a full-duplex directional collision avoidance protocol (FDDCA) [17], in which communication nodes are equipped with directional transducers that can freely adjust the activated sector. However, only one sector can be activated at any given time. The Non-Orthogonal Multiple Access (NOMA) protocol, which enables multiple users to share the channel over the same time, frequency, and spatial resources, effectively increases network capacity and improves spectral efficiency to some extent [18,19]. However, the omnidirectional multi-user broadcasting mechanism lacks advantages in covert communication.

With the advancement of array technology and signal processing capabilities, directional underwater acoustic communication is widely regarded as a key technology to enhance the capacity and reliability of underwater acoustic networks. Through beamforming and sectorized communication, nodes can focus acoustic energy toward a target direction, effectively improving the link signal-to-noise ratio (SNR) and suppressing interference from non-target directions. In multi-node network scenarios, directional communication further evolves into multi-directional or spatially multiplexed communication, allowing the same time-frequency resources to be reused by multiple spatially separated links. Some studies have focused on the impact of link asymmetry and directionality on network performance in underwater acoustic communication, and have introduced directional communication and directed graph modeling concepts at the MAC or routing layer to improve medium access and link scheduling performance [20,21]. However, these works primarily focus on the communication process itself, with relatively little attention paid to the system modeling and protocol design for node discovery under directional transmission conditions. Although some studies have attempted to integrate the node discovery process with physical layer signal structures or cross-system communication mechanisms [22,23], the use of spatial awareness information to improve the reliability and efficiency of node discovery in directional underwater acoustic communication scenarios remains an area that requires further investigation. Zhang et al. proposed a neighbor discovery protocol (MDT-ND) with multi-directional antennas, in which each node is equipped with a multi-directional transducer capable of both omnidirectional and multi-directional transmission and reception. This allows the simultaneous transmission or reception of multiple data streams. By dynamically adjusting the transmission power and detecting the distance to neighboring nodes, the protocol effectively reduces collisions and enhances communication efficiency. However, the protocol does not provide a detailed discussion on the localization of neighboring nodes [MDT-ND] [15].

Under directional communication conditions, beam alignment errors can significantly degrade the reliability of link transmission. To effectively mitigate the communication performance degradation caused by beam misalignment, it is crucial to obtain high-precision spatial distribution information between underwater nodes in real-time. To this end, Direction of Arrival (DOA) estimation and Ultra-Short Baseline (USBL) positioning technologies have been widely applied in underwater sensing and localization [24,25,26] but have not yet been integrated into a closed-loop discovery protocol. Existing studies, from various information fusion perspectives, have proposed joint localization methods based on Received Signal Strength (RSS), Time Difference of Arrival (TDOA), and azimuth angle measurements and have systematically analyzed their observability conditions and error bounds [27,28]. Relevant research indicates that by integrating multiple physical layer measurements, spatial awareness accuracy can be significantly improved under complex underwater channel conditions [29,30]. Furthermore, addressing issues such as multipath propagation, signal-to-noise ratio fluctuations, and limited array scale in underwater environments, researchers have proposed various USBL localization methods based on azimuth angles or phase differences [31,32] and have verified their effectiveness under full ocean depth, sensor errors [33], and non-line-of-sight propagation conditions [34]. However, existing research on DOA and USBL primarily focuses on improving positioning accuracy and algorithm performance, with applications mostly limited to target tracking or navigation tasks. There is still a lack of in-depth studies on how to systematically integrate spatial awareness results into the underwater communication networking process, particularly in the node discovery and access initialization stages.

The detection capability of the proposed DBCB protocol can be used to obtain the direction of arrival (DOA) and the source distance, enabling the localization of neighboring nodes. By demodulating the signal, prior information of neighbors can be acquired, thereby accelerating the neighbor discovery process. Simulation results demonstrate that the DBCB protocol efficiently and quickly completes the neighbor node discovery task. By combining directional communication, space-time-frequency multiplexing, OFDM technology, and localization and bearing estimation functions, it provides an efficient and reliable discovery mechanism for underwater acoustic communication networks in complex marine environments. This is particularly suitable for rapid network formation and efficient communication initialization in underwater unmanned platform clusters. The main contributions of this study are as follows:

- Directional communication enables parallel multi-beam discovery, reducing interference and improving discovery success by focusing signals in specific directions;
- The adaptive power control mechanism adjusts transmission power based on node distance and azimuth, enhancing low power consumption and interference resilience;
- By integrating signal detection and azimuth estimation, the system dynamically schedules beam resources for precise alignment, improving topology stability and network performance;
- The efficiency, robustness, and feasibility of the protocol are validated through an anechoic tank experiment, providing reliable data for future field trials.

The structure of the remaining sections is organized as follows: Section 2 presents a detailed system modeling of the underwater acoustic link, laying the foundation for the proposed protocol. Section 3 describes the principles of the proposed protocol, including the key concepts and algorithms involved, as well as the associated azimuth estimation method. Section 4 provides an evaluation of the proposed protocol’s performance through both simulation results and anechoic tank experiments, demonstrating its effectiveness and robustness in real-world conditions. Finally, Section 5 concludes the paper, summarizing the main findings and highlighting potential areas for future research.

## 2. Underwater Acoustic Link Model

This study establishes an underwater acoustic channel model that comprehensively considers propagation loss, array directivity, and multipath effects. The proposed model consists of three major components: large-scale attenuation, spatially selective array gain, and small-scale multipath and scattering effects. It is designed to describe the propagation characteristics of underwater acoustic signals under specific transceiver configurations. In shallow water environments, for a signal with frequency *f*, the propagation loss of the signal when traveling through an underwater acoustic channel over a distance *d* is typically expressed as

(1) $$A\left(d,f\right)=k\cdot 10log_{10}\left(d\right)+d\cdot \alpha \left(f\right)$$

where *d* is the propagation distance, *k* is the geometric spreading factor. The parameter *k* is determined by the geometric characteristics of underwater acoustic propagation. When acoustic waves propagate under ideal spherical spreading conditions, k=2; under ideal cylindrical spreading conditions, k=1. In practical underwater acoustic channels, the value of *k* typically lies between 1 and 2. As a compromise between these two extremes, we set k=1.5 in this study. α(f) is the frequency-dependent absorption coefficient, expressed in decibels (dB), which is modeled empirically. The absorption coefficient α(f) for a frequency *f* (in kHz) is given by the following expression

(2) $$\alpha \left(f\right)=0.1\frac{f^{2}}{1+f^{2}}+40\frac{f^{2}}{4100+f^{2}}$$

In underwater acoustic channels, multipath propagation is a common phenomenon where the transmitted signal reaches the receiver via multiple propagation paths. Let $d_{n}$ denote the length of the *n*-th propagation path, where n=1,2,…,N. The channel transfer function, accounting for the contributions from each propagation path, is expressed as

(3) $$H\left(d,f\right)=\sum_{n=1}^{N}\frac{\Phi_{n}}{\sqrt{A(d_{n},f)}}e^{-j(2\pi f\tau_{n}\left(d\right)+\phi_{n})}$$

where $\Phi_{n}$ represents the additional loss on the *n*-th path, such as reflection loss, and $A(d_{n},f)$ is the propagation loss for the *n*-th path, which depends on the distance $r_{n}$ and the frequency *f*. The propagation delay for the *n*-th path is given by $\tau_{n}\left(d\right)=d_{n}/c$, where *c* is the standard underwater sound speed. The term $\phi_{n}$ is the random phase offset for the *n*-th path, reflecting the phase shift introduced by the multipath effects. Equation (3) captures the combined influence of all propagation paths, including their delays, losses, and phase shifts, providing a comprehensive model of the underwater acoustic channel.

Different from the traditional omnidirectional point source assumption, the stereophonic circular array transducer used in this study exhibits significant directional characteristics at both the transmitter and receiver, resulting in a distinct spatial selectivity in the channel gain. The effective received signal power depends not only on the propagation distance but also on the actual departure and arrival angles of the signal, as well as the relative alignment between the selected sectors of the transmitter and receiver arrays. Let $\theta_{t}$ and $\theta_{r}$ denote the true departure and arrival angles, respectively, while $\theta_{s,t}$ and $\theta_{s,r}$ represent the normal directions of the selected sectors at the transmitter and receiver, respectively. The energy attenuation caused by beam misalignment can be expressed as

(4) $$\left\{\begin{array}{c} G_{tx}\left(\theta_{t}\right)={\left|AF\left(\theta_{t}-\theta_{s,t}\right)\right|}^{2}\\ G_{rx}\left(\theta_{r}\right)={\left|AF\left(\theta_{r}-\theta_{s,r}\right)\right|}^{2} \end{array}\right.$$

where AF(·) is the normalized array factor function, approximated based on a continuous linear array. This modeling approach effectively captures the scalloping loss caused by beam misalignment and explicitly couples the beam selection strategy at the physical layer with variations in link SNR, providing a unified interface for subsequent directional transmission and cross-layer simulation analysis.

When an underwater acoustic signal propagates from the transmitter to the receiver, it experiences various attenuation and interference effects. The received power $P_{rx}$ is the effective signal power received at the receiver, which depends on the transmitted power $P_{tx}$, the array gains $G_{tx}$ and $G_{rx}$, and the channel transfer function H(d,f). The channel transfer function H(d,f) describes the attenuation and phase shift of the signal as it propagates through the channel. In this context, the received power $P_{rx}$ can be expressed as

(5) $$P_{rx}=P_{tx}\cdot G_{tx}\left(\theta_{t}\right)\cdot G_{rx}\left(\theta_{r}\right)\cdot {\left|H\left(d,f\right)\right|}^{2}$$

where $G_{tx}$ and $G_{rx}$ represent the array gains at the transmitter and receiver, respectively, in the corresponding directions. H(d,f) is the channel transfer function, which captures the combined effects of signal attenuation, propagation path, and noise factors. The magnitude ${\left|H\left(d,f\right)\right|}^{2}$ reflects the signal’s attenuation and phase shift as it propagates through the channel, serving as a critical indicator of the energy loss encountered during propagation.

The Signal-to-Noise Ratio (SNR) is a key parameter for quantifying the quality of the signal. SNR represents the ratio of signal power to noise power. At the receiver, if the noise power is denoted as $P_{N}$, the SNR can be expressed as

(6) $$SNR=\frac{P_{rx}}{P_{N}}=\frac{P_{tx}\cdot G_{tx}\left(\theta_{t}\right)\cdot G_{rx}\left(\theta_{r}\right)\cdot {\left|H\left(d,f\right)\right|}^{2}}{P_{N}}$$

The environmental noise N(f) in underwater acoustic channels is typically assumed to be white noise, with its power spectral density being flat over a specific frequency range. The noise power $P_{N}$ within the frequency range $\left[f_{c}-B/2,f_{c}+B/2\right]$ can be expressed as

(7) $$P_{N}=\int_{f_{c}-B/2}^{f_{c}+B/2}N\left(f\right)df$$

where $f_{c}$ is the center frequency and *B* is the bandwidth of the system. This integral represents the total noise power within the given frequency range, assuming the noise is evenly distributed across the bandwidth.

The equivalent channel gain $H_{e}$ represents the change in signal gain due to factors such as propagation loss, frequency-selective fading, and multipath effects after the signal has passed through the channel. To simplify calculations, the equivalent power gain of the channel is defined as

(8) $$|H_{e}{|}^{2}=G_{tx}\left(\theta_{t}\right)\cdot G_{rx}\left(\theta_{r}\right)\cdot {\left|H\left(d,f\right)\right|}^{2}$$

Considering the relationship between the received power $P_{rx}$ and the equivalent gain $H_{e}$, we can further simplify the expression for the received power. Therefore, the received power $P_{rx}$ can be simplified as

(9) $$P_{rx}=P_{tx}\cdot {\left|H_{e}\right|}^{2}$$

The SNR is a key metric for assessing communication quality. By introducing the equivalent gain $H_{e}$, the calculation of received power becomes simpler, facilitating performance analysis under varying channel conditions. The SNR can be expressed as

(10) $$SNR=\frac{P_{tx}\cdot {\left|H_{e}\right|}^{2}}{P_{N}}\geq \gamma_{\min}$$

This redefinition of the SNR better accounts for the channel transfer characteristics, especially in underwater acoustic communication, where channel gain and multipath propagation significantly impact signal quality. By considering $|H_{e}{|}^{2}$, we can more accurately assess the actual signal quality of the communication link.

**Figure 1:**
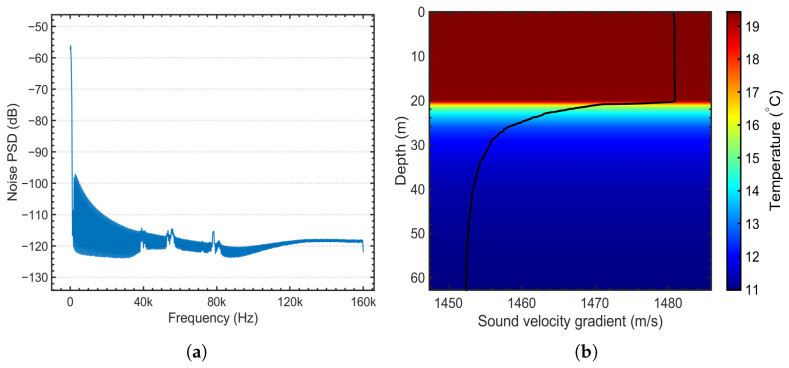
Underwater noise power spectral density and sound velocity profiles in Qiandao Lake. (**a**) Noise power spectral density. (**b**) Sound velocity profile.

For the transmitted power $P_{t}$, the received signal power at a distance d is given by ${\left|H\left(d,f\right)\right|}^{2}$. However, due to the random components in the channel gain, it is not possible to precisely adjust the transmit power. Therefore, we define the nominal transmit power required for a link at distance $r_{0}$ as

(11) $$P_{t}=P_{0}+\eta \cdot Pe$$

where $P_{0}$ represents the radio consumption of the circuit, η is the acoustic-to-electric conversion efficiency, and $P_{e}$ is the input power. Therefore, we can further derive the following

(12) $$Pe\geq \frac{P_{N}\cdot \gamma_{\min}-P_{0}\cdot {\left|H_{e}\right|}^{2}}{\eta \cdot |H_{e}{|}^{2}},\left(0<\eta \leq 1\right)$$

where $P_{N}$ is the noise power in the communication channel, and $\gamma_{\min}$ is the minimum required SNR, ensuring reliable communication and successful signal detection at the receiver. The underwater power spectral density and sound velocity profile are shown in Figure 1.

The performance of underwater acoustic communication systems depends on the receiver’s ability to maintain a sufficiently high signal-to-noise ratio. SNR is influenced by factors such as ambient noise, the directional gain of the equipment, propagation losses, and the frequency-dependent characteristics of the channel. Even with constant source power, the observed SNR at the receiver can vary significantly with frequency and distance due to the medium’s inherent absorption and attenuation characteristics.

## 3. Protocol System Principles

This work aims to develop a neighbor discovery protocol that achieves high efficiency in both completion time and discovery accuracy, while enabling reliable neighbor localization. The protocol is designed for a star-shaped topology comprising a master node and multiple subordinate nodes, and ensures network connectivity between any two participating nodes under power constraints.

### 3.1. Neighbor Discovery

Our objective is to design a neighbor discovery protocol that achieves high efficiency in both completion time and discovery performance while enabling accurate node localization. The proposed protocol operates within a star-shaped network topology consisting of a master node and multiple subordinate nodes. Under power constraints, the protocol ensures reliable network connectivity between any two participating nodes. The communication model is established under the following assumptions:

- The network consists of N nodes, and the master node is aware of the total number of nodes *N*. All nodes are uniformly distributed within the same depth layer. Each node is equipped with a directional transceiver of limited bandwidth and a beamwidth of θ=2π/K, where *K* is an integer.
- The communication coverage of each node is divided into *K* non-overlapping fan-shaped sectors, each of which is considered a standard sector for analytical simplicity. Nodes employ a directional transmission and omnidirectional reception mode, assuming ideal beam alignment and negligible beamforming errors.
- All nodes operate in a half-duplex mode, meaning that a node can either transmit or receive at any given time but not both simultaneously. Communication between a pair of nodes occurs only when one node is in the transmit state, the other is in the receive state, and their distance is within the communication range.
- Each node is assigned a unique identifier (ID) and can adjust its transmission power between a minimum $P_{\min}$ and a maximum $P_{\max}$, ensuring consistent communication coverage at different power levels. Each node possesses *M* available subcarriers, from which one subcarrier is randomly selected for data transmission in each communication attempt.

**Figure 2:**
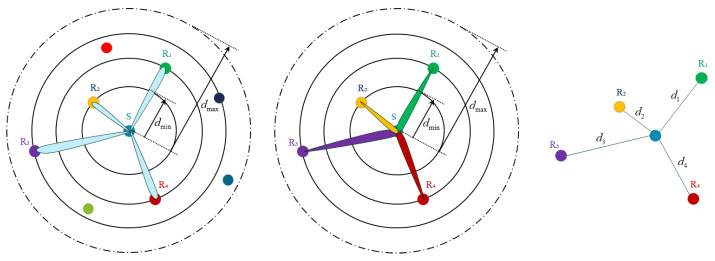
Nodes are randomly located within a circular area.

These assumptions form the foundation of the proposed protocol design, supporting efficient neighbor discovery, robust connectivity, and location-aware communication under the physical constraints of underwater acoustic networks. The AUV cluster formation task consists of one master node and *N* subordinate nodes. Each node is equipped with an *m*-path directional transceiver, capable of parallel transmission and reception. This allows the master node to simultaneously transmit different data streams to all subordinate nodes using beam-based directional transmission. The network is deployed within a circular area of radius *R*, as shown in Figure 2.

In conventional underwater acoustic networks, subordinate nodes typically use omnidirectional transducers that radiate energy uniformly in all directions, limiting the effective communication range. In a communication–sensing integrated architecture, the master node employs a directional transducer with beamwidth Θ, concentrating transmit power within the beam and thus extending both communication and sensing ranges under the same power conditions. Given the sound speed in water *c*, the propagation delay τ=d/c grows linearly with distance *d*. Therefore, the neighbor discovery process must address time-slot misalignment and feedback asynchrony caused by long propagation delays.

During neighbor discovery, the master node periodically broadcasts a Hello probe signal over the entire coverage area in each discovery cycle. Upon receiving the probe, subordinate nodes decide, based on predefined rules, whether to reply with a Response signal. The transmission signal is denoted as $T_{j}^{i}$ and the received signal as $R_{j}^{i}$, where i and j represent the frequency and beam indices, respectively. Here, i∈[1,M],j∈[1,K], and o=0xFF; i=o represents any frequency band and j=o denotes all beams. The basic operations are defined as $send(T_{j}^{i},pkt^{TP_{n}})$, $listen(R_{o}^{o},pkt^{TP_{n}})$, and $recv(T_{j}^{i},pkt^{TP_{n}})$, forming the foundation for coordinated signal exchange between the master and subordinate nodes.

Let the master node be denoted by *S*, and the set of subordinate nodes be represented as $R=\{R_{1},R_{2},\ldots ,R_{N}\}$. When a subordinate node $R_{i}$ successfully receives the Hello signal $H(t_{k})$ transmitted by *S* at time $t_{k}$, it determines based on its internal state and the received signal content whether to transmit a Reply signal $P_{i}\left(t_{k}\right)$. If the unique identifier (ID) of $R_{i}$ already exists in the neighbor set $N(t_{k})$ maintained by the master node, $R_{i}$ does not send a reply and remains in the listening state. Otherwise, if its ID is not included in $N(t_{k})$, it immediately sends a Reply signal and then switches back to the listening mode. If a node fails to receive any Hello signal within a discovery cycle, it continuously remains in the listening state without initiating any feedback. Upon receiving a Reply signal from $R_{i}$, the master node extracts several signal parameters, including the propagation delay $\tau_{i}$, angle of arrival $\theta_{i}$, received signal-to-noise ratio $SNR_{i}$, arrival time $T_{i}^{\mathrm{arr}}$, received power $P_{i}^{\mathrm{rx}}$, and possible Doppler shift $\Delta f_{i}$. Based on these parameters, the master node estimates the distance $d_{i}=c\tau_{i}$, bearing information, and link quality metrics of $R_{i}$. The node information is then recorded as a feature vector $V_{i}\left(t_{k}\right)=\left\{ID_{i},d_{i},\theta_{i},SNR_{i},P_{i}^{rx},State_{i}\right\}$ for subsequent analysis and network maintenance. Subsequently, the discovered node is added to the neighbor set as

(13) $$N\left(t_{k+1}\right)=N\left(t_{k}\right)\cup \left\{V_{i}\left(t_{k}\right)\right\}$$

To improve the discovery coverage of undetected nodes, if there still exist nodes $R_{j}\notin N\left(t_{k}\right)$, the master node adjusts its transmit power in the next cycle according to $P_{S}\left(t_{k+1}\right)=f\left(P_{S}\left(t_{k}\right)\right)$ and continues transmitting Hello signals to expand the detection range. The master node S serves as the center of the network, with subordinate nodes randomly distributed around it. Communication between nodes occurs within a range defined by the minimum communication distance $d_{\min}$ and the maximum communication distance $d_{\max}$. The nodes are assigned a set of power levels G={1,2,…,G}, where each node has a finite number of power levels. Each power level g∈G corresponds to a specific communication distance $d_{g}$, which is related to the minimum distance $d_{\min}$ and the interval Δd between power levels. Specifically, the communication distance $d_{g}$ is given by $d_{g}=d_{0}+g\Delta d$, where $d_{0}=d_{\min}$ represents the minimum communication distance and Δd is the distance interval between power levels. The maximum communication distance $d_{\max}$ corresponds to the power level G−1, and can be expressed as

(14) $$d_{G-1}=d_{\max}=d_{0}+\left(G-1\right)\Delta d$$

Given that the network contains N nodes, the node density is defined as $\rho =N/\pi R^{2}$, where R is the radius of the node distribution. The gradient Δd between power levels represents the change in communication distance corresponding to each power level, and is calculated using the formula:

(15) $$\Delta d=\frac{d_{\max}-d_{\min}}{G-1}$$

where G represents the total number of power levels. Based on the parameters of our custom communication prototype, we set G=32. By adjusting the number of power levels, different communication ranges can be achieved to meet varying network requirements. From Equation (10), it follows that to ensure the receiver correctly decodes the signal from the transmitter, the signal-to-noise ratio at the receiver must exceed a certain decoding threshold $\gamma_{\min}$. Therefore, the minimum threshold transmission power for transmitting node-*i* can be expressed as:

(16) $$p_{i}^{*}\geq \frac{P_{N}\cdot \gamma_{\min}}{G_{tx}\left(\theta_{t}\right)\cdot G_{rx}\left(\theta_{r}\right)\cdot {\left|H\left(d_{i},f\right)\right|}^{2}}$$

We denote ϖ as the maximum number of concurrent links at the same time, with ϖ≤min(k,m), The optimal matched transmission power can be obtained by quantizing $p_{i}^{*}$ into 32 discrete levels while ensuring that the total power satisfies $\sum_{i=1}^{\varpi }p_{i}^{*}\leq P_{\max}$. The quantized power for level $g_{*}$ is given by:

(17) $$g_{*}=\frac{\left(p_{i}^{*}-P_{\min}\right)\left(G-1\right)}{P_{\max}-P_{\min}}+1,g_{*}\in G$$

**Figure 3:**
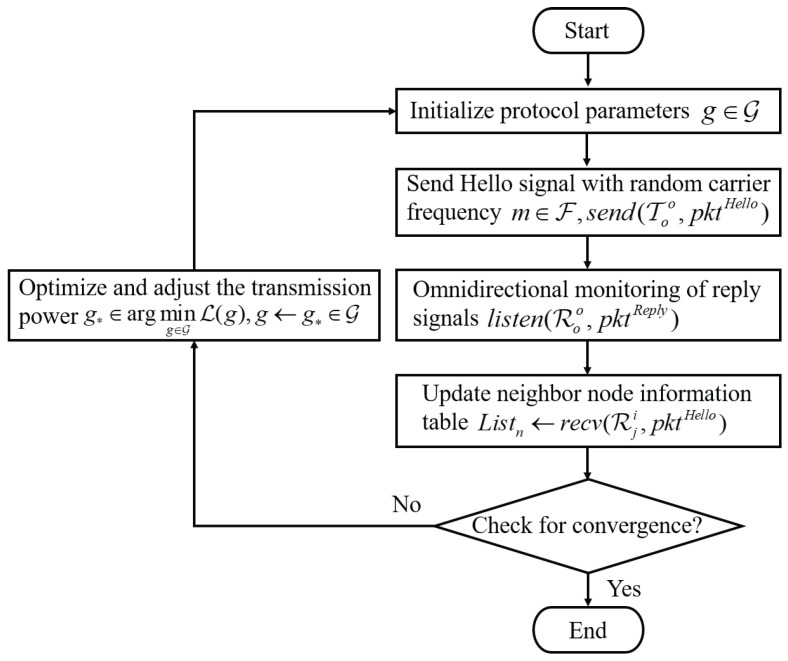
Flowchart of master node discovery during the priori exploration phase.

This configuration allows for more refined control over node communication ranges, enabling better network management and optimization. In practical applications, the configuration of power levels and node density plays a crucial role in determining the network’s performance, coverage, and communication reliability. During each information exchange, both the master and subordinate nodes update their neighbor tables in real time to ensure the timeliness and consistency of N(t). Through the updated neighbor information model, each node can adaptively adjust its transmission power based on the power control function, enabling effective channel resource allocation and energy optimization. Through the above neighbor discovery and power adaptation mechanisms, the protocol enables efficient neighbor detection and network initialization in dynamic underwater environments, providing reliable network connectivity assurance for subsequent communication phases, as shown in Figure 3. When the neighbor discovery set satisfies $|N(t_{k})|=N$ or the discovery period reaches its predefined upper limit, the master node terminates the omnidirectional discovery phase and subsequently switches to the directional multi-beam validation phase.

**Figure 4:**
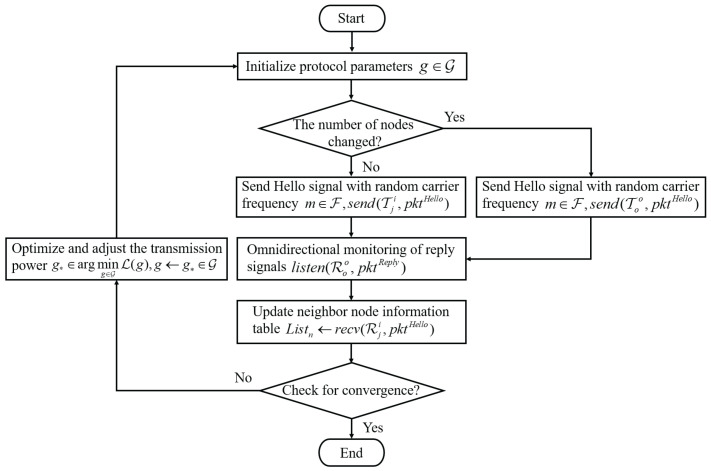
Flowchart of master node discovery during the directional exploration phase.

During the discovery process, the master node utilizes an a priori neighbor information table $T={\left\{ID_{i},p_{i},f_{i}^{*},\eta_{i}\right\}}_{i=1}^{N}$, where ${\mathrm{ID}}_{i}$ represents the node identifier, $p_{i}$ denotes the historical position estimate, $f_{i}^{*}$ indicates the optimal operating frequency band, and $\eta_{i}$ corresponds to the channel adaptation metric. Based on the information in T, the master node preconfigures the beamforming direction $\theta_{k}$ and bandwidth allocation $M_{k}$. It then employs orthogonal frequency-division multiplexing (OFDM) to generate ${\left\{B_{k}\left(t\right)\right\}}_{k=1}^{N}$ independent, non-interfering beams, with each beam assigned a unique center frequency $f_{k}$. Based on the preliminary neighbor information obtained from prior discovery, the master node groups the neighbors in the set $N_{0}=\left\{n_{1},n_{2},\ldots ,n_{|N_{0}|}\right\}$. Specifically, the master node partitions the neighbors into several subsets according to their distance, direction, or frequency preference, and then initiates a directional discovery task using a frequency-divided, multibeam transmission mode. According to the frequency assignment, initial direction estimation, and channel quality prediction recorded in T, the master node selects a set of frequency indices $F=\{f_{1},\ldots ,f_{M}\}$ and a set of beam directions $\Theta =\{\theta_{1},\ldots ,\theta_{K}\}$, and preconfigures the beamforming direction $\theta_{k}$ and bandwidth allocation $B_{k}$ for each beam. Using orthogonal frequency-division multiplexing (OFDM), the master node generates multiple independent, non-interfering beams, with each beam assigned a unique center frequency $f_{k}$. A mapping function $G_{\ell }\left(f_{i},\theta_{j}\right)$ is then applied to assign each neighbor a unique frequency–beam pair. Subsequently, the master node begins to transmit Hello probing signals simultaneously in multiple frequency bands, directed towards all target directions, as illustrated in Figure 4. In terms of frequency allocation, the DBCB protocol combines directional and omnidirectional modes to collaboratively complete the network node discovery task. For a given number of subordinate nodes N, the master node performs flexible dynamic resource allocation and optimization with the goal of maximizing the link spectral efficiency ($L_{se}$).

(18) $$\arg\max_{f_{d_{i}}}\;L_{se}\left(f_{c_{i}};f_{d_{i}};r_{i}\right),\;s.t.\;\sum_{i=1}^{N}f_{d_{i}}\leq f_{B},f_{c_{i}}\propto \frac{1}{r_{i}}$$

where $r_{i}$ denotes the distance between subordinate node-*i* and the master node, $f_{ci}$ represents the center frequency of the band allocated to subordinate node-*i*, $f_{di}$ denotes the bandwidth occupied by node-*i*, and $f_{B}$ is the total available bandwidth of the underwater acoustic network system.

The subordinate nodes detect valid Hello frames based on energy detection and select the operating frequency band according to the signal-to-noise ratio (SNR) threshold $\gamma_{\min}$. The response strategy follows a dual-mode mechanism: when $\gamma_{\min}>12\;\mathrm{dB}$, the node replies using the frequency band $f^{*}$ assigned by the master node (Mode A); when $\gamma_{\min}\leq 12\;\mathrm{dB}$, it randomly selects a frequency $f_{r}$ from the predefined set F to transmit the Reply signal (Mode B), thereby increasing the probability of successful reporting under low-SNR conditions. The Reply frame includes the node identifier (ID), channel state information (CSI) vector $h_{i}$, and the round-trip delay estimate $\tau_{i}$, and is encoded using Turbo coding to enhance transmission reliability.

The master node adopts a multi-channel parallel reception mechanism to simultaneously process Reply signals arriving from different beams and frequency bands. Based on the received feedback, it dynamically adjusts the receiver gain $G_{\mathrm{rx}}$ and the transmit power $G_{\mathrm{tx}}$ of subsequent Hello frames. Successfully discovered neighbor nodes are recorded in the dynamic neighbor information table $T_{D}$. Specifically, when a neighbor node $n_{k}$ receives a Hello message, it determines its uplink reply frequency band according to the frequency index $f_{i}$ carried in the message header. The node then either selects the corresponding frequency band $f_{i(k)}$ or randomly chooses an available frequency from the candidate set to transmit its reply. Subsequently, it sends the Reply signal along its local beamforming direction. With the support of the multibeam receiving array, the master node can simultaneously receive multiple Reply signals from different nodes. The master node performs matched filtering and energy detection across all beam–frequency combinations to obtain the set of responding neighbors ${\hat{N}}_{0}$. It then compares ${\hat{N}}_{0}$ with the a priori neighbor list $N_{0}$ to identify the unresponsive nodes U. If |U|>0, the master node reinitiates discovery by adjusting the transmit frequency bands or enhancing the beamforming gain in the corresponding directions, based on the previously recorded channel parameters $\left(f_{i(k)},\theta_{k},{\mathrm{SNR}}_{k}\right)$. If unresponsive nodes remain after frequency adjustment, the master node further refines the beam set from $\Theta \to \Theta^{\prime }$ to improve directional alignment and continues the detection process. This iterative band adjustment–beam reconstruction–rediscovery procedure continues until the termination condition is satisfied, i.e., |U|=0 or the number of iterations reaches the predefined threshold $\sigma_{\max}$. Once all neighbor nodes are stably detected, the discovery phase concludes. This mechanism achieves precise neighbor detection, adaptive frequency selection, and link state evaluation, thereby establishing a reliable foundation for subsequent directional link formation and network communication.

### 3.2. Frame Format and Scheduling Strategy

The underwater nodes described in this study are equipped with multi-directional transducers, enabling simultaneous transmission and reception of multiple signals. The transducer arrays at both the transmission and reception terminals establish multiple independent, non-interfering bidirectional exclusive channels. Leveraging the structural advantages of the transducers, the neighbor discovery protocol can be designed using a combination of omnidirectional and directional transmission. This approach allows for efficient node discovery over large areas and long distances. The neighbor discovery protocol consists of two types of information frames: the detection frame (Hello) and the reply frame (Reply). The detection frame is sent by the initiating node to request the establishment of a link, while the reply frame serves as feedback from the receiving node in response to the detection frame.

**Figure 5:**
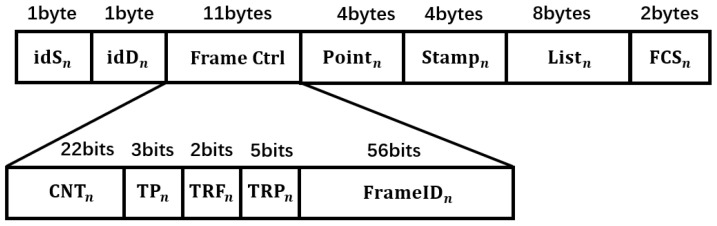
The structure and information of the control frame.

The control frame structure is shown in Figure 5. The field ${\mathrm{idS}}_{n}$ represents the sender node’s ID, and ${\mathrm{idD}}_{n}$ represents the receiver node’s ID. A new value for ${\mathrm{Cnt}}_{n}$ is generated with each transmission of a Hello packet. The field ${\mathrm{TP}}_{n}$ indicates the type of the detection packet generated by node n; when this field is 0, it indicates a Hello packet, and when it is 1, it indicates a Reply packet. The field ${\mathrm{trF}}_{n}$ represents the transmission frequency band number, ${\mathrm{trP}}_{n}$ represents the transmission power, and ${\mathrm{FrameID}}_{n}$ is the frame sequence number (reserved field). ${\mathrm{Point}}_{n}$ contains the three-dimensional location information of node *n*, ${\mathrm{tStamp}}_{n}$ indicates the transmission timestamp of the packet, ${\mathrm{List}}_{n}$ is the list of neighboring nodes, and ${\mathrm{FCS}}_{n}$ represents the frame’s checksum for error detection. This design, featuring short control frame length and high information density, is particularly well-suited for underwater acoustic communication channels, which typically experience low data rates, high delays, and high error rates. It significantly reduces protocol overhead while enhancing network robustness and the efficiency of topology maintenance.

During the actual operation of the system, signal transmission and processing experience varying degrees of delay, which are typically deterministic and can be obtained through experimental or theoretical analysis methods. Therefore, the following section explains how time information can be accurately obtained during the signal reception and transmission processes.

The time required for the signal to travel from the completion of MAC layer encapsulation to the transducer transmission is defined as the transmission response delay $D_{s}$. This delay consists of several parts: The DBCB protocol unit reads the local time $\tau_{t}$, then writes the local time into the packet and sends it to the modem via the serial port, with a time delay of $\tau_{t_{1}}$. The modem parses the packet and generates the digital signal to be transmitted, taking $\tau_{t_{2}}$. The digital transmit signal is then transmitted to the front-end preprocessing unit via the EMIF interface, with a delay of $\tau_{t_{3}}$. The front-end preprocessing unit performs operations such as digital-to-analog conversion, with a delay of $\tau_{t_{4}}$. Finally, the analog signal is output to the power amplifier and then to the transducer, with a negligible delay of $\tau_{t_{5}}$. Therefore, the accurate transmission time of the underwater acoustic signal $D_{s}$ (transmission response delay) is given by

(19) $$D_{s}=\tau_{t}+\tau_{t_{1}}+\tau_{t_{2}}+\tau_{t_{3}}+\tau_{t_{4}}$$

The time duration of an underwater acoustic communication signal for one MAC frame is defined as the transmission signal duration $T_{s}$. Therefore, the total transmission time after the Hello signal is encapsulated by the MAC layer is denoted as

(20) $$T_{send}=D_{s}+T_{s}$$

The time at which the underwater acoustic signal reaches the array is the reception time of the signal. However, during the uplink transmission of the underwater acoustic signal, there is a reception delay. Therefore, the MAC layer needs to estimate the actual time at which the array receives the signal based on the transmission delay. After the node receives the underwater communication signal and successfully demodulates it, the DBCB protocol responds to the received signal. Once the master node finishes transmitting the RTS signal, it immediately switches to the listening state. The listening time of the master node under the DBCB protocol is denoted as

(21) $$T_{listen}=2T_{t}-T_{s}+2D_{r}+D_{s}$$

where $D_{r}$ represents the reception response delay, and $T_{t}$ is the propagation time of the underwater acoustic signal in the water. The underwater acoustic signal is sampled at the array in the form of data blocks, with a sampling rate of $f_{s}$ and a block length of $L_{s}$. Assuming the preamble of the underwater acoustic signal is sampled within a data block, and its starting position is at the *Q*-th point in the data block, this introduces a delay $\tau_{r_{1}}=\left(L_{s}-Q\right)/f_{s}$. Considering the continuity of data processing, the data block needs to be delayed and shifted for storage, meaning the current arriving data block is processed after a delay of one block time, which introduces a delay $\tau_{r_{2}}=L_{s}/f_{s}$. The processing time for detecting the preamble is $\tau_{r_{3}}$. After detecting the preamble, the signal processing unit sends the stored underwater acoustic signal data block to the modem, with a transmission time of $\tau_{r_{4}}$, which can be ignored. Upon receiving the underwater acoustic signal data block, the modem immediately sends the starting position information of the preamble to the network protocol unit via the serial port, taking $\tau_{r_{5}}$. After the network protocol unit receives the starting position information, it immediately reads the local system time $\tau_{r}$. Thus, the accurate reception time $D_{r}$ (reception response delay) is given by

(22) $$D_{r}=\tau_{r}-\tau_{r_{1}}-\tau_{r_{2}}-\tau_{r_{3}}-\tau_{r_{5}}$$

After the master node’s DBCB protocol listens for $T_{listen}$ time, it receives the CTS signal feedback from the slave node. To ensure the completeness of the master’s listening period, an additional signal protection time $T_{p}$ is added, where the waiting time is set according to the propagation delay corresponding to the distance to the target. Specifically, the time required to complete the transaction at a power level of *l* is given by

(23) $$T_{l}=T_{send}+T_{listen}+T_{p}$$

**Figure 6:**
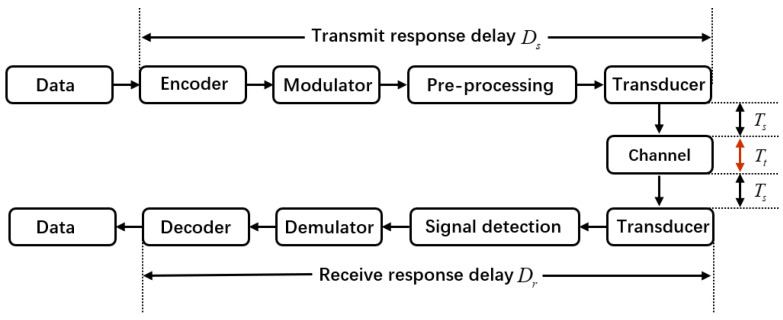
Illustrates the data flow and delay information during the signal transmission and reception process.

The transmission and reception of the Hello packet involve operations at both the data link layer and the physical layer. The data flow and operation delay information during the signal transmission and reception process are shown in Figure 6. Once the network communication task begins, the DBCB protocol continues to run in the background, although its speed may be reduced. This approach facilitates a recovery process to prevent node loss, particularly in dynamic or mobile environments. This adaptive mechanism improves overall network performance, ensuring that communication remains uninterrupted even in environments with fluctuating coverage or node mobility. By integrating this recovery process, the DBCB protocol enhances the stability and reliability of the network, ensuring it can effectively handle challenges in mobile and dynamic scenarios.

### 3.3. DOA Estimation Method

In the detection phase, the far-field source signal is incident on the array, and the deviation between the incident angle and the normal axis of the array elements results in significant amplitude variations. This provides a solid physical foundation for azimuth estimation based on Received Signal Strength (RSS). Simultaneously, the spatial position differences between array elements introduce small Time Difference of Arrival (TDOA), enabling high-resolution azimuth estimation using the Ultra-Short Baseline (USBL) principle. This study adopts a multimodal estimation strategy combining both RSS and TDOA, integrated with an energy-guided constraint fusion method. During the coarse localization phase, RSS is used to lock the target sector range, while in the fine localization phase, the RSS estimate is incorporated as prior information within a Adaptive Constrained Weighted Least Squares (AC-WLS) framework. This approach ensures the robustness of RSS in low Signal-to-Noise Ratio (SNR) conditions while leveraging the accuracy of TDOA in high-quality channels, thereby achieving both robustness and precision in the spatial awareness of communication networks.

**Figure 7:**
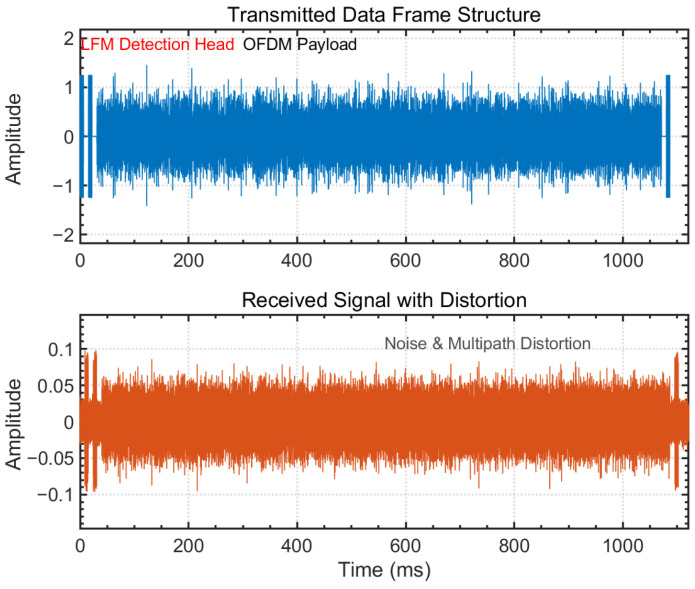
OFDM signal frame structure.

To achieve robust initial azimuth estimation, this study leverages the asymmetric distribution of received energy across the array elements of a stereophonic circular array and employs a nonlinear least squares fitting method based on beamforming model matching. It is assumed that the transmitting node emits a frame of signals modulated using Orthogonal Frequency Division Multiplexing (OFDM), as shown in Figure 7. After propagating through the underwater acoustic channel and undergoing attenuation due to the directional characteristics of the array elements, the received power in each channel of the receiver array follows the theoretical beam pattern of the transducer for signal reception.

The header of the OFDM signal frame embeds a Linear Frequency Modulated (LFM) pulse as a probing signal. The received power is extracted via matched filtering, where the time–bandwidth product of the LFM signal is exploited to obtain processing gain. This enables the system to effectively suppress sonar pulses or noise that do not match the LFM signal parameters, thereby enhancing the detection capability of each channel. At the same time, it minimizes multipath interference, providing high-quality input data for sector-level Sinc function fitting and reducing the false alarm rate. Specifically, the time-domain signal received by the *k*-th array element can be expressed as

(24) $$R_{k}\left(t\right)=A_{k}\cdot S\left(t-\tau_{k}\right)+N_{k}\left(t\right)$$

where S(t) denotes the transmitted LFM preamble signal, $A_{k}$ is the signal amplitude modulated by the directional gain, $\tau_{k}$ is the propagation delay, and $N_{k}\left(t\right)$ represents additive white Gaussian noise. To extract the signal energy from noise, a cross-correlation operation is performed at the receiver

(25) $$X_{c,k}\left(t\right)=\int_{-\infty }^{+\infty }R_{k}\left(\tau \right)S^{*}\left(\tau -t\right)d\tau$$

The integral operation in this process performs a correlation between the received signal and the complex conjugate of the transmitted signal. Essentially, this is a process of determining the match between the received and transmitted signals, using convolution to enhance the detectability of the signal amidst noise. Subsequently, by searching for the maximum squared magnitude of the correlation function $X_{c,k}\left(t\right)$, the peak power $P_{\mathrm{meas},k}$ of the signal can be extracted. The peak power is expressed as

(26) $$P_{\mathrm{meas},k}=\max_{t}{\left|X_{c,k}\left(t\right)\right|}^{2}$$

The significance of this operation lies in determining the signal’s energy by searching for the maximum value of the correlation function $X_{c,k}\left(t\right)$. Since signal energy is often difficult to directly identify in the presence of noise, the method of searching for the maximum squared magnitude effectively separates the useful signal from noise and accurately estimates the signal’s strength. The observed power $P_{\mathrm{meas},k}$ is defined as the maximum power value of the envelope of the correlation function, expressed as follows

(27) $$P_{\mathrm{meas},k}={\left(\max_{t}\left|\int R_{k}\left(\tau \right)S^{*}\left(\tau -t\right)d\tau \right|\right)}^{2}$$

**Figure 8:**
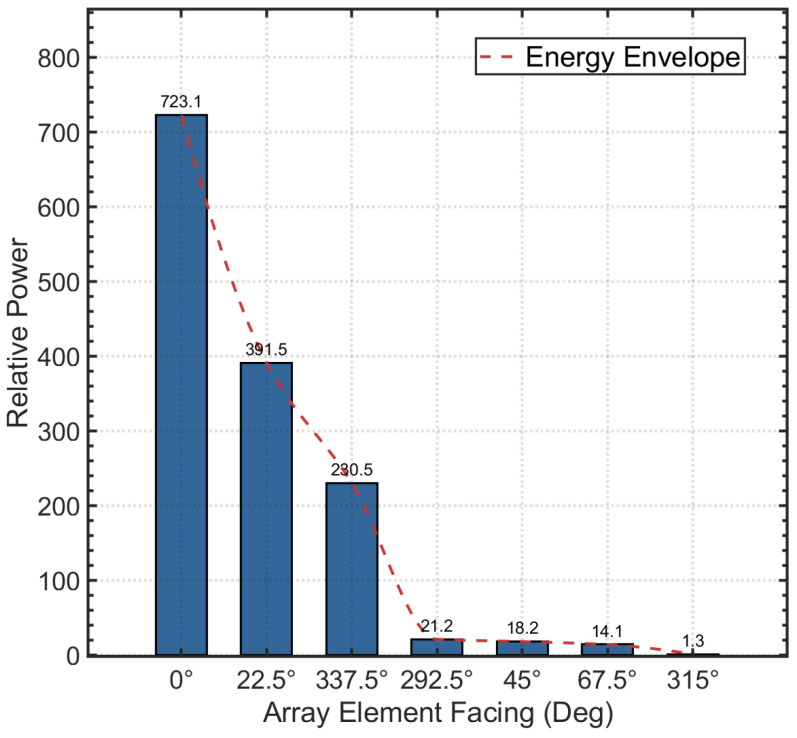
Spatial energy distribution characteristics of the stereophonic circular array after receiving far-field signals.

This formulation ensures reliable power estimation for subsequent azimuthal sector fitting under low-SNR and multipath-prone underwater acoustic environments.The spatial energy distribution characteristics of the array receiving signals are shown in Figure 8.

After extracting the per-channel energy features $P_{\mathrm{meas},k}$ via matched filtering, this study exploits the directional diversity of the elements in the stereophonic circular array by adopting a beam pattern matching algorithm based on Nonlinear Least Squares (NLS) to achieve high-accuracy initial azimuth estimation.

Considering the unavoidable presence of additive white Gaussian noise in the received signals, the measured energy $P_{\mathrm{meas},k}$ is in practice a superposition of the signal envelope and the noise floor. To compensate for beam pattern distortion under low signal-to-noise ratio (SNR) conditions, the following theoretical observation model incorporating a noise power compensation term ${\hat{P}}_{n}$ is established:

(28) $$\hat{P}\left(\phi ,P_{0}\right)=P_{0}\cdot {\left(\frac{sin(\Psi_{k})}{\Psi_{k}}\right)}^{2}+{\hat{P}}_{n}$$

where ϕ denotes the target azimuth angle to be estimated, $P_{0}$ represents the equivalent peak signal power, and ${\hat{P}}_{n}$ is the estimated in-band noise power. The variable $\Psi_{k}$ characterizes the angular relationship between the incident wave direction and the element normal, and is defined as

(29) $$\Psi_{k}=\frac{\pi L}{\lambda }\cdot sin\left(\phi -\alpha_{k}\right)$$

where L represents the array length, λ being the acoustic wavelength, and $\alpha_{k}$ denoting the physical orientation angle of the *k*-th array element. This formulation enables robust initial azimuth estimation by jointly accounting for element directivity and noise effects.

The algorithm selects the seven array elements with the highest received energy to form a local observation set K. Since the axial directivity of the transducers follows the radiation model of a continuous linear array, the theoretical power gain of the *k*-th element can be represented by a Sinc function. To estimate the optimal azimuth angle ϕ and signal strength $P_{0}$, the following cost function is constructed as the sum of squared residuals between the measured power and the theoretical model:

(30) $$J\left(\phi ,P_{0}\right)=\sum_{k\in K}{\left(P_{\mathrm{meas},k}-\left[P_{0}\cdot {\left(\frac{sin(\Psi_{k})}{\Psi_{k}}\right)}^{2}+{\hat{P}}_{n}\right]\right)}^{2}$$

To achieve sector-level estimation accuracy, the Sequential Quadratic Programming (SQP) algorithm is employed to jointly optimize the parameter vector $x={\left[\phi ,P_{0}\right]}^{T}$. The SQP-based joint optimization of the azimuth angle ϕ and signal power $P_{0}$ allows $P_{0}$ to be treated as a free variable, thereby adaptively compensating for amplitude fluctuations caused by propagation loss and ensuring accurate azimuth estimation:

(31) $$\left[{\hat{\phi }}_{RSS},{\hat{P}}_{0}\right]=arg\min_{\phi ,P_{0}}J\left(\phi ,P_{0}\right)$$

Furthermore, by constraining the search space to a $\pm 11.25^{\circ }$ neighborhood around the normal direction of the dominant array element, the proposed method effectively avoids local minima induced by transducer sidelobes. Owing to its strong nonlinear search capability, the SQP algorithm is able to capture subtle gradients in the beam envelope, thereby overcoming the limitations imposed by the discrete distribution of physical array elements and achieving sector-level localization accuracy. From a practical implementation perspective, the computational complexity of the proposed SQP optimization is extremely low and well-suited for underwater nodes with constrained processing power. The optimization involves only two state variables ($N_{var}=2$: azimuth ϕ and peak power $P_{0}$) and operates on a localized observation set of K=7 elements. Consequently, the dimension of the Hessian matrix in the SQP subproblem is merely 2 × 2, reducing matrix operations to constant time complexity O(1). By using the physical orientation of the array element with the strongest received signal as the initial estimate, the search space is highly localized, allowing the algorithm to typically converge within only $N_{iter}$ iterations. The overall complexity scales at $O(K\cdot N_{iter})$, requiring minimal floating-point operations that can be efficiently executed in real-time by modern embedded processors.

After obtaining the coarse azimuth estimate from the beam model fitting, the rough azimuth ${\hat{\phi }}_{\mathrm{RSS}}$ is converted into a unit direction vector in three-dimensional space. Assuming the target is in the far field with an elevation angle $\theta \approx 0^{\circ }$, the direction vector is defined as

(32) $$u_{r}={\left[cos\left({\hat{\phi }}_{RSS}\right)cos\left(\hat{\theta }\right),sin\left({\hat{\phi }}_{RSS}\right)cos\left(\hat{\theta }\right),sin\left(\hat{\theta }\right)\right]}^{T}$$

In the TDOA spatial observation model, the system selects the *J* array elements with the highest received energy as a local observation subset. The element with the maximum received energy is denoted as $P_{\mathrm{ref}}$, and the remaining auxiliary elements are $P_{j},j=2,3,\ldots ,J$. Under the far-field planar wave assumption, the direction vector u and the TDOA observations $\Delta \tau_{j}$ satisfy the linear relationship $\left(P_{j}-P_{\mathrm{ref}}\right)\cdot u=c\cdot \Delta \tau_{j}$, where *c* is the speed of sound in water. To suppress interference from low-SNR off-axis elements, a diagonal weighting matrix $W=\mathrm{diag}(w_{2},\ldots ,w_{J})$ is introduced. According to the Cramér-Rao lower bound (CRLB), the variance of time-delay estimates is inversely proportional to the linear SNR. For directional arrays, the SNR of each channel satisfies $SNR_{l,i}\propto {\left|AF\left(\phi -\alpha_{i}\right)\right|}^{2}$, leading to the final definition of weights $w_{i}\propto {\left|AF\left(\phi -\alpha_{i}\right)\right|}^{2}$, where $\alpha_{i}$ is the physical orientation of the *i*-th element. To optimally fuse RSS priors with TDOA observations, an augmented matrix is constructed to convert azimuth estimation into a constrained optimization problem. Based on the minimum variance unbiased estimation (MVUE) theory, the cross-modal constraint factor λ satisfies $\lambda \propto \sigma_{\mathrm{TDOA}}^{2}/\sigma_{\mathrm{RSS}}^{2}$. Considering the inverse relationship between TDOA variance and linear SNR, we set $\lambda =1/SNR_{l,0}$ dynamically. The direction vector u is obtained via a weighted least-squares solution of the augmented system:

(33) $$\left[\begin{array}{c} \sqrt{W}A\\ \sqrt{\lambda }I \end{array}\right]u=\left[\begin{array}{c} \sqrt{W}b\\ \sqrt{\lambda }u_{r} \end{array}\right]$$

where the *j*-th row of matrix A is ${\left(P_{j}-P_{\mathrm{ref}}\right)}^{T}$, and the *j*-th element of b is $c\cdot \Delta \tau_{j}$. After normalization, the azimuth can be extracted as $\hat{\phi }=\arctan2\left(u_{y},u_{x}\right)$.

Due to the physical overlap between adjacent array elements, even if the target lies at the intersection of two beams, it remains within the −3 dB beamwidth, ensuring that the base signal energy is reliably captured. The system does not rely on precise physical beam alignment but instead effectively resolves the off-axis angle in continuous space by combining the received signal strength (RSS) envelope and time difference of arrival (TDOA) measurements. One limitation of this study is the assumption of uniform deployment depth. The cylindrical array provides horizontal directivity, but significant depth variation introduces elevation angles, thereby reducing the array’s effective horizontal gain. Future work will explore optimizing the transducer’s vertical directivity and develop a three-dimensional joint azimuth-elevation algorithm to accommodate underwater networks with depth variations.

## 4. Analysis of Simulation and Tank Test Results

### 4.1. Simulation Results Analysis

We validated the performance of the DBCB protocol through semi-physical simulations, anechoic tank experiments, and lake trials. The anechoic tank experiment plays a crucial role in the development and performance evaluation of underwater acoustic communication systems, offering significant technical and engineering advantages. The anechoic tank provides a stable and controllable propagation environment, allowing precise control of experimental variables and ensuring repeatable results. Experiments conducted in this environment accurately reflect the performance limits of the system under ideal conditions. Additionally, the anechoic tank effectively reduces multipath effects and background noise, making it ideal for testing communication signal propagation characteristics. By comparing experimental results with simulations, propagation models and system parameters are calibrated, enabling a more accurate assessment of system performance. This environment supports multi-beam transmission and reception experiments involving a master node and multiple slave nodes, allowing for comprehensive testing of interactions between spatial reuse, multiple access, power control, beam alignment, and scheduling algorithms. The repeated experiments and data collection provide valuable insights into the protocol’s stability, interference resistance, and energy efficiency under various configurations, offering foundational verification and risk assessment for subsequent lake trials in more complex environments.

**Figure 9:**
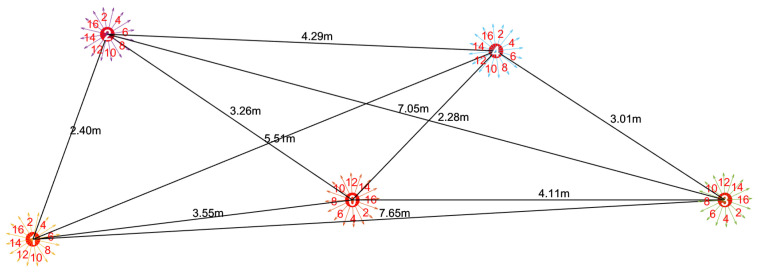
Schematic diagram of the deployment positions and distances of the five simulated nodes.

**Figure 10:**
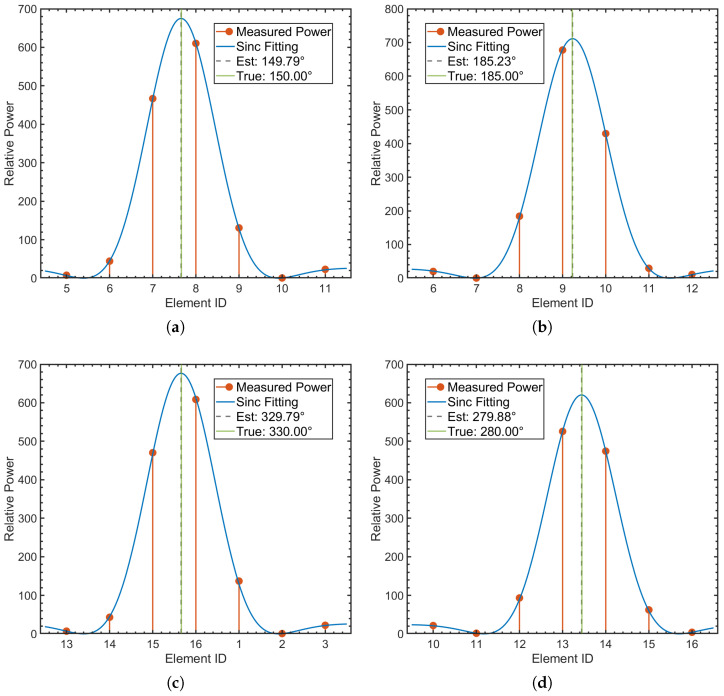
Azimuth estimation numerical simulation of the 4 received signals by the master node. (**a**) Azimuth estimate for Node 1. (**b**) Azimuth estimate for Node 2. (**c**) Azimuth estimate for Node 3. (**d**) Azimuth estimate for Node 4.

Before conducting the anechoic tank experiments, we first performed system-level simulations to verify the feasibility of the proposed DBCB protocol and the effectiveness of its scheduling strategy. To validate the effectiveness of the proposed DOA estimation and directional beam switching algorithm in underwater acoustic communication, we designed a simulation experiment aimed at modeling signal transmission from source nodes located at different azimuths, and performing Direction of Arrival (DOA) estimation and beam matching based on array received signals. In the experiment, we employed a sixteen-element array with a misaligned distribution, and simulated source nodes transmitting signals at random azimuth angles ranging from 0° to 360°. The signals were modulated using Differential Quadrature Phase Shift Keying (DQPSK) and transmitted via Orthogonal Frequency Division Multiplexing (OFDM) to facilitate parallel communication. To validate the estimation accuracy of the proposed algorithm within the omnidirectional coverage range, numerical simulations of azimuth estimation were conducted on the star topology nodes shown in Figure 9. During the simulation, the master node received signals from four distinct subordinate nodes through its array to estimate their azimuth angles. Figure 10a–d present the energy distribution fitting curves of these four subordinate nodes on the master node array, reflecting the reception characteristics of the signals at different azimuths.

During signal reception, we used the array-received signal data to perform DOA estimation using the least squares method. Through array signal processing, we calculated the direction of arrival (DOA) of the signal. After obtaining the DOA estimate, we combined it with the geographic azimuth information provided by a compass and matched it with the known azimuth angle by minimizing the error between the DOA estimate and the predefined beam direction, ensuring accurate signal reception. To validate the accuracy of the algorithm, we compared the DOA estimates with the actual source azimuths and calculated the estimation errors. Select the appropriate Array Element (SAE) based on the beamforming configuration and identify its corresponding Beam ID (BID) to define the direction. Record the Physical Orientation of the Element (POE) and measure the Signal Incidence Angle (SIA). Calculate the Linear Array Factor (LAF) and Signal Attenuation Value (SAV) to assess array sensitivity and signal strength loss, as shown in Table 1. The numerical simulation results show that the system can accurately capture the main lobe envelope of the beam at different azimuths, demonstrating the high precision of the algorithm in practical applications. Specifically, for Node 1 (true azimuth 150°), the estimated value is 149.77°, with an absolute error of only 0.23°; for Node 2 (true azimuth 185°), the estimated value is 184.83°, with an absolute error of 0.17°; for Node 3 (true azimuth 330°), the estimated value is 329.86°, with an error of only 0.14°; and for Node 4 (true azimuth 280°), the estimated value is 280.31°, with an absolute error of 0.31°. These results indicate that the proposed algorithm consistently maintains excellent estimation accuracy across various azimuths.

**Figure 11:**
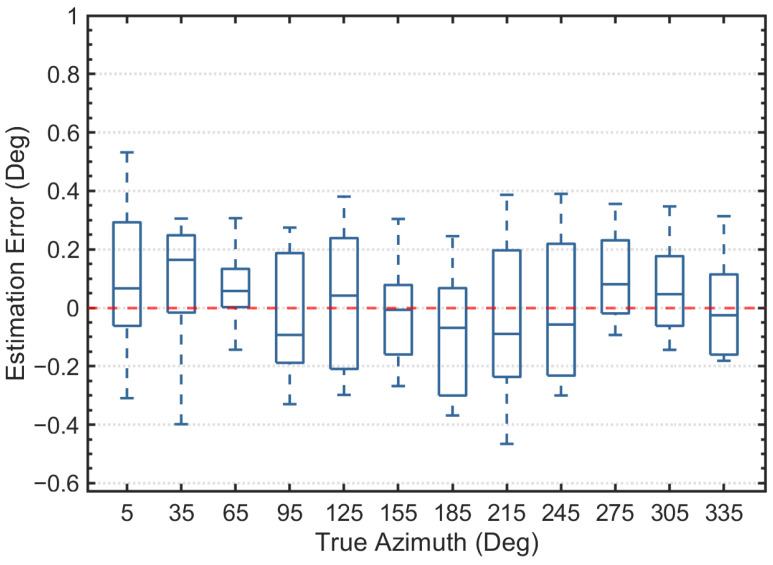
Statistical distribution of azimuth angle estimation Error.

We have experimentally validated the high accuracy and robustness of the proposed protocol for DOA estimation and beam matching in underwater acoustic communication. From the statistical results, whether the target is located at the center of the sector or at the overlapping edge of two array elements, the RSS-based estimation algorithm, which utilizes the Sinc model fitting, demonstrates outstanding linearity and stability. Even in complex signal environments, this algorithm provides high-precision azimuth angle estimation, ensuring the reliability of the system. Figure 11 presents a box plot showing the distribution of azimuth estimation errors in the simulation. For all sampled azimuth angles, the median error closely aligns with the 0° reference line, with the average absolute error in azimuth angle being only 0.21°. Additionally, the interquartile range (IQR) for all angles remains within a small interval, and the overall absolute maximum error is confined to approximately ±0.6°, indicating that the algorithm does not exhibit significant systematic bias under these simulation conditions. The error distribution data objectively demonstrate that the algorithm maintains convergence in numerical solving, even in scenarios with random noise and multipath interference, without experiencing large-scale localization divergence. This highlights the algorithm’s high stability and low error. These characteristics make it highly promising for practical applications, especially in underwater acoustic communication systems that require high-precision positioning and azimuth awareness.

Subsequently, we performed a systematic simulation to verify the scheduling strategy and performance of the DBCB. The simulations aimed to assess key performance indicators of the protocol under various network scales, transmission power levels, and the number of multi-beam transmissions, including the network node discovery rate, average discovery delay, and energy efficiency. Through the quantitative analysis of these metrics, we provide theoretical foundations and parameter references for the subsequent physical experiments. The simulation platform was implemented using a combined MATLAB (Version: R2022a) and C++ (Version: VC++2022) environment. The simulation scenario was a 3D underwater communication environment, with a water depth set to 30 m. All nodes were placed at the same depth to minimize the effects of vertical sound refraction. The underlying channel model was based on the Bellhop ray-tracing propagation model, which accurately simulates multipath effects, path loss, seawater absorption, and noise characteristics. The channel impulse response and sound speed profile have been described in Section 2.

The DBCB protocol achieves efficient discovery and reliable localization of neighbor nodes in underwater acoustic networks by integrating multi-beam parallel directional access and azimuth estimation algorithms, providing the foundational support for subsequent network communication. The system bandwidth is set to 10 kHz, with a center frequency spacing of 10 kHz, corresponding to a channel available rate of 3.4 kbps, which aligns with the typical parameters of a medium-range underwater acoustic communication system. The network topology follows a star structure, consisting of one master node and N slave nodes. The slave nodes are randomly and uniformly distributed within a 2.5 km radius of the master node. Each node has multi-beam transceiver capability (1≤K≤16) and is equipped with M = 4 available carrier frequency resources. The master node can activate up to 4 beams simultaneously for parallel communication. The simulations evaluate three key performance indicators: node discovery rate, node discovery delay, and energy consumption. The simulation results are compared with two mechanisms, directional transmission and reception (DTR) and random two-way (RTW) [16], under different sector conditions in terms of node discovery rate and average node discovery delay.

**Figure 12:**
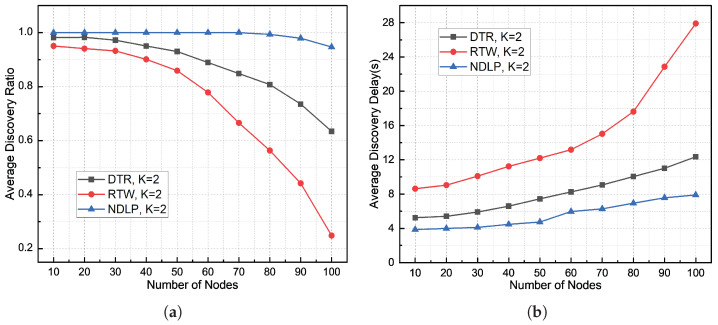
Impact of node count on the performance of different neighbor discovery mechanisms. (**a**) Comparison of node discovery rates among the three mechanisms when K = 2. (**b**) Comparison of node discovery delays among the three mechanisms when K = 2.

**Figure 13:**
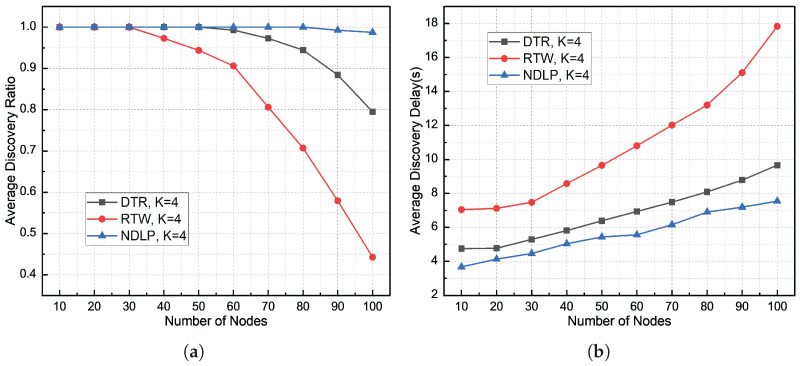
Impact of node count on the performance of different neighbor discovery mechanisms. (**a**) Comparison of node discovery rates among the three mechanisms when K = 4. (**b**) Comparison of node discovery delays among the three mechanisms when K = 4.

**Figure 14:**
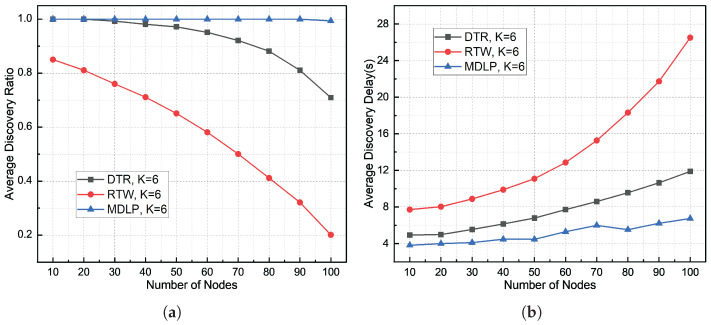
Impact of node count on the performance of different neighbor discovery mechanisms. (**a**) Comparison of node discovery rates among the three mechanisms when K = 6. (**b**) Comparison of node discovery delays among the three mechanisms when K = 6.

Figure 12, Figure 13 and Figure 14 illustrate the comparison of node discovery efficiency and average discovery delay across different protocols when the number of beam sectors varies. From the node discovery rate statistics in these three figures, it can be observed that when the network density is low, the node discovery rates of both the DTR and RTW mechanisms remain relatively high. As the node density increases, the discovery rates of both mechanisms begin to significantly decline. However, the node discovery rate of the proposed DBCB protocol remains high. This is because the proposed protocol employs a silence mechanism for already discovered nodes, meaning that if a slave node receives a Hello signal and the corresponding node ID is already in the neighbor table, the slave node will not send a Reply signal, thus avoiding collisions. From the discovery delay statistics in the three figures, it is evident that as the number of nodes in the network increases, the average node discovery delay for the DBCB protocol, as well as for the DTR and RTW mechanisms, increases. However, the discovery delay of the DBCB protocol increases at a significantly lower rate compared to the DTR and RTW mechanisms. This is because the proposed protocol’s parallel multi-beam transmission and reception mechanism avoids the time overhead associated with polling and effectively reduces signal collisions.

The experimental results in Figure 12a show that when K = 2, the average node discovery rate of the DTR mechanism is 87.32%, the average node discovery rate of the RTW mechanism is 72.82%, and the average node discovery rate of the DBCB protocol is 99.2%. As the node scale increases, the node discovery rate of all three protocols decreases. Specifically, the DTR mechanism decreases by 34.73%, the RTW mechanism decreases by 70.22%, and the DBCB protocol’s node discovery rate decreases by 5.31%. Figure 12b shows that the average node discovery delay for the DTR mechanism is 8.13 s, for the RTW mechanism is 14.78 s, and for the DBCB protocol is 5.58 s. As the node scale increases, the node discovery delay for all three protocols increases. The DTR mechanism increases by 7.11 s, the RTW mechanism increases by 19.29 s, and the DBCB protocol increases by 4.04 s. The experimental results show that the DTR and RTW mechanisms are more sensitive to changes in node scale. As the network size increases, the performance of both the DTR and RTW mechanisms declines significantly, especially when node density is high. These mechanisms are more susceptible to collisions and interference, leading to a significant reduction in node discovery rates and an increase in discovery delays. The performance degradation of the DTR mechanism is primarily due to its reliance on omnidirectional signal propagation and time-slot allocation, which in high-density environments often causes signal overlap and collisions, thereby impacting network throughput and stability. Although the RTW mechanism reduces collisions through randomized communication, as the node scale increases, the randomness fails to effectively mitigate signal conflicts, resulting in a decrease in node discovery efficiency.

The experimental results in Figure 13a show that when K = 4, the average node discovery rate of the DTR mechanism is 95.89%, the average node discovery rate of the RTW mechanism is 83.58%, and the average node discovery rate of the DBCB protocol is 99.8%. As the node scale increases, the node discovery rate of all three protocols decreases. Specifically, the DTR mechanism decreases by 20.5%, the RTW mechanism decreases by 55.75%, and the DBCB protocol’s node discovery rate decreases by 1.25%. Figure 13b shows that the average node discovery delay for the DTR mechanism is 6.8 s, for the RTW mechanism is 10.89 s, and for the DBCB protocol is 5.61 s. As the node scale increases, the node discovery delay for all three protocols increases. The DTR mechanism increases by 4.91 s, the RTW mechanism increases by 10.79 s, and the DBCB protocol increases by 3.87 s. The DBCB protocol addresses collision issues more effectively as the node scale increases by utilizing multi-beam transmission and dynamically adjusting beam directions. With the increase in the number of available beams, the DBCB protocol can enhance network throughput and node discovery efficiency through parallel transmission and spatial reuse, even with limited time-frequency resources. Once a node is identified and its ID is added to the neighbor table, if the slave node receives a Hello signal again, it will no longer send a Reply signal, thereby avoiding redundant transmissions. As the number of available beams increases, the advantages of the DBCB protocol in terms of node discovery performance become even more pronounced compared to the DTR and RTW mechanisms.

The experimental results in Figure 14a show that when K = 6, the average node discovery rate of the DTR mechanism is 92.21%, the average node discovery rate of the RTW mechanism is 58.01%, and the average node discovery rate of the DBCB protocol is 99.94%. As the node scale increases, the node discovery rate of all three protocols decreases. Specifically, the DTR mechanism decreases by 29.03%, the RTW mechanism decreases by 64.94%, and the DBCB protocol’s node discovery rate decreases by 0.63%. Figure 14b shows that the average node discovery delay for the DTR mechanism is 7.68 s, for the RTW mechanism is 14.02 s, and for the DBCB protocol is 5.06 s. As the node scale increases, the node discovery delay for all three protocols increases. The DTR mechanism increases by 6.95 s, the RTW mechanism increases by 18.8 s, and the DBCB protocol increases by 2.92 s. The detailed changes in node discovery rate and average discovery delay are shown in Table 2 and Table 3. The increase in the number of available beams enables the DBCB protocol to effectively utilize spatial resources, significantly enhancing communication capacity in large-scale environments. By adopting multi-beam transmission, the DBCB protocol ensures that the same time-frequency resources can be reused across multiple spatial channels, thereby reducing network congestion and the likelihood of collisions. The DBCB protocol also introduces a silence mechanism for already discovered nodes, avoiding redundant signal transmissions and further reducing collisions and interference in the network. By precisely controlling the transmission direction and timing for each node, the protocol ensures high efficiency and low latency during communication. This makes the DBCB protocol exhibit significant performance advantages in large-scale node environments, particularly in dense and complex scenarios, where it can maintain a high node discovery rate and low discovery delay.

**Figure 15:**
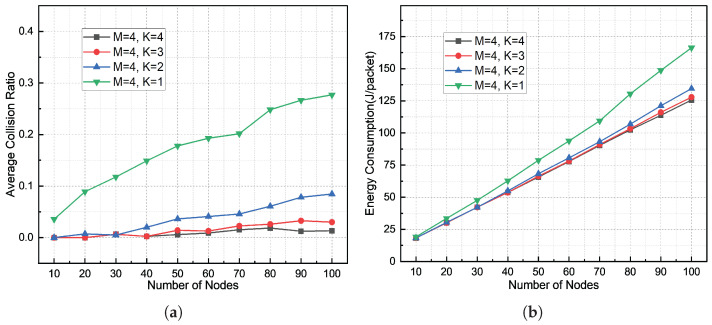
Collision rate and energy consumption statistics for the DBCB protocol. (**a**) Variation of collision rate with the number of nodes under different beam numbers. (**b**) Variation of energy consumption with the number of nodes under different beam numbers.

Figure 15 presents the collision rate and energy consumption statistics for the DBCB protocol. Figure 15a shows the variation of collision rate with the number of nodes under different beam numbers. As the node scale increases, the collision rate rises accordingly. When M = 1, 2, 3, and 4, the average collision rates are 17.55%, 3.78%, 1.47%, and 0.83%, respectively, indicating that the multi-beam parallel directional transmission mechanism is sensitive to the number of available beams. Figure 15b shows the variation of energy consumption with the number of nodes under different beam numbers. As the node scale increases, the network energy consumption also increases. When K = 1, 2, 3, and 4, the average energy consumption per data packet is 89.05 J, 75.07 J, 72.74 J, and 71.98 J, respectively. Increasing the number of beams improves node discovery efficiency through parallel transmission and spatial reuse. This allows the network to handle larger scales and higher node densities, enhancing throughput and discovery rates. Importantly, this efficiency improvement does not result in higher energy consumption. The DBCB protocol reduces redundant transmissions and collision-induced energy waste through multi-beam transmission and a silence mechanism for discovered nodes, ensuring optimal energy consumption.

### 4.2. Anechoic Tank Test Results Analysis

**Figure 16:**
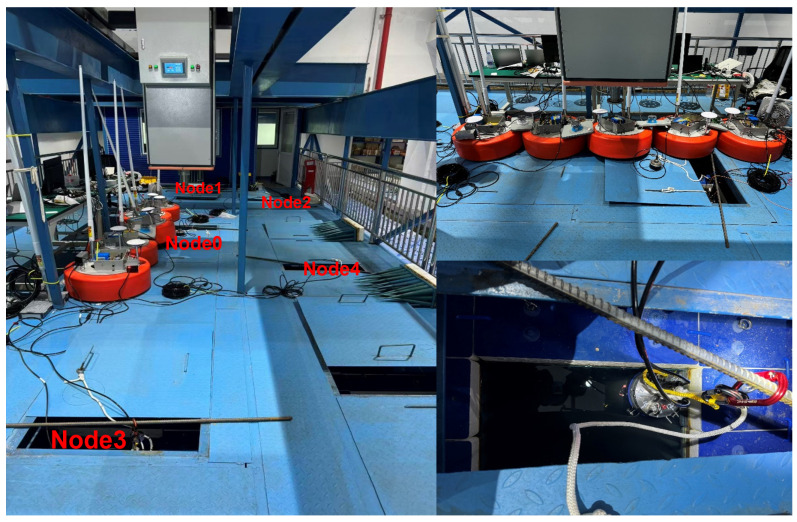
Schematic diagram of the anechoic tank experimental site environment.

The simulation results have thoroughly validated the effectiveness and stability of the DBCB protocol in multi-node dynamic underwater acoustic networks. However, the simulation environment still involves certain idealized assumptions, such as simplified channel models, weakened multipath effects, and uniform noise characteristics, which differ from real-world underwater acoustic environments. To further evaluate the protocol’s performance and engineering feasibility under actual acoustic conditions, we conducted system validation experiments based on the simulation study, utilizing an anechoic tank. The anechoic tank provides a stable and controllable experimental environment, effectively eliminating external interference and echo reflections, thus facilitating the verification and analysis of the protocol’s key performance metrics. We performed real-time underwater acoustic communication and networking system tests in the anechoic tank, as shown in Figure 16. The tank dimensions are 13.1 m × 7.7 m × 3.5 m, with wedge-shaped sound-absorbing structures on the walls and floor, achieving a sound absorption coefficient ≥90% (1 kHz∼2 kHz) and ≥99% (2 kHz∼100 kHz). This setup effectively absorbs sound wave reflections, ensuring that one-way propagation conditions approximate free-field conditions. The water temperature is maintained at 22 ± 0.5 °C, the salinity is 0‰, and the sound speed is stable at approximately 1498 m/s. In this study, a controllable point-to-multipoint communication experimental platform was established in the laboratory’s anechoic tank to validate the feasibility and performance of the proposed DBCB protocol and multi-beam directional communication mechanism in a real-world environment. The deployment positions of the nodes in the anechoic tank are shown in Figure 16.

To evaluate the signal detection and direction-of-arrival (DOA) estimation performance under multi-frequency concurrent conditions, this study designed an anechoic tank experiment based on multi-carrier concurrent signal detection and beam matching. The experiment used four independent frequency sub-bands (denoted as $f_{1},f_{2},f_{3},f_{4}$), each with a bandwidth of 10 kHz and a center frequency spacing of 10 kHz. The master node simultaneously transmits independent data streams to the four slave nodes, with valid communication signals present on each sub-band. To ensure the independence of signal separation and detection, the signals on each frequency are transmitted synchronously in time, with different pseudo-random code modulation used for each band to facilitate subsequent signal separation and correlation detection. The experimental results show that the angular error of beam matching is controlled within ±2.3°. By calculating the received signal power, signal-to-noise ratio (SNR), and bit error rate (BER) for each beam, the results indicate that under the four-frequency concurrent conditions, the average SNR of each signal is 13.2 dB, 12.8 dB, 14.1 dB, and 13.6 dB, with the BER remaining below the threshold. This demonstrates that the system, operating in a multi-beam and multi-frequency concurrent mode, is capable of stable detection of multiple signals, precise DOA estimation, and rapid beam matching.

**Figure 17:**
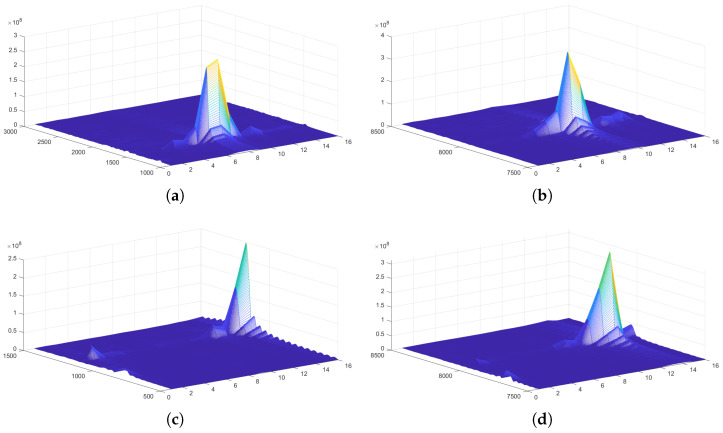
Signal detection results in four frequency bands at the receiver of the anechoic tank experiment. (**a**) Detection results in Frequency Band $f_{1}$: Maximum beam is 8. (**b**) Detection results in Frequency Band $f_{2}$: Maximum beam is 9. (**c**) Detection results in Frequency Band $f_{3}$: Maximum beam is 16. (**d**) Detection results in Frequency Band $f_{4}$: Maximum beam is 14.

In the anechoic tank, we conducted practical communication experiments using multiple communication nodes. During the overall testing process, the system collected array data in 1-min intervals. Within each 1-min period, multiple communication events targeting different nodes typically occurred, resulting in multiple synchronized headers across different frequency bands. To analyze the performance of the signal detection module, the sampled data was input into a frequency-domain digital pulse compression framework based on Linear Frequency Modulation (LFM) signals. The detection results are shown in Figure 17. When a synchronized header is detected, the detection framework records its position and peak value within the entire data segment, while also providing threshold lines for the forward and backward noise segments. As shown in the figure, the frequency-domain digital pulse compression framework based on LFM signals demonstrated effective signal decision-making in the anechoic tank, with no false alarms or missed detections. This result confirms the robustness and accuracy of the proposed detection method in real-world water acoustic environments, ensuring reliable signal detection in complex scenarios.

**Figure 18:**
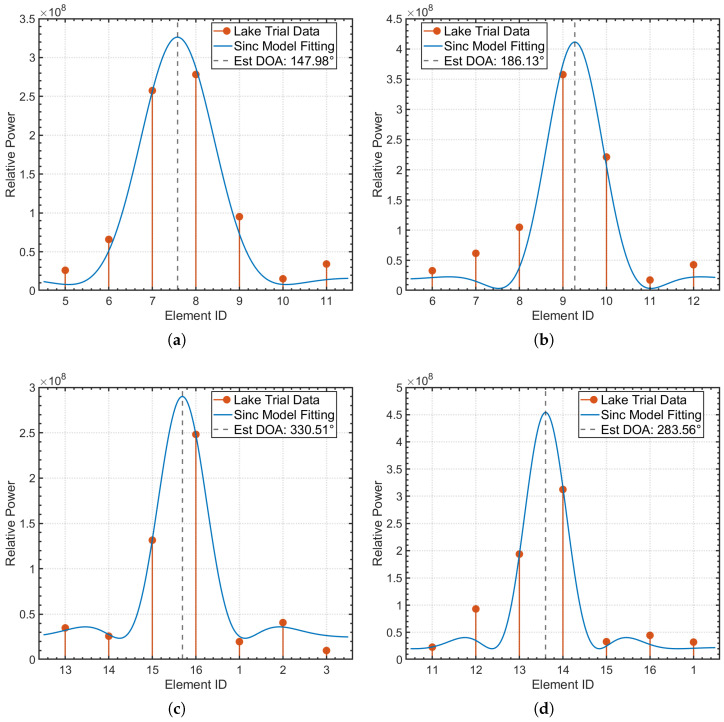
Energy distribution and beam azimuth fitting curves for four subordinate nodes in the anechoic tank experiment. (**a**) Beam azimuth fitting for Frequency Band $f_{1}$: 147.98°. (**b**) Beam azimuth fitting for Frequency Band $f_{2}$: 186.13°. (**c**) Beam azimuth fitting for Frequency Band $f_{3}$: 330.51°. (**d**) Beam azimuth fitting for Frequency Band $f_{4}$: 283.56°.

The direction of arrival (DOA) of a signal can be initially determined by the main beam of the signal peak, and a more accurate estimation can be obtained by analyzing the time delay of the array signal peaks. Therefore, the DOA estimation can be divided into two steps: First, beam power fitting is performed based on the signal power; second, DOA estimation is carried out using the peak time delays of multiple signals. By performing second-order polynomial fitting on the beam power, an initial estimate of the signal’s direction is obtained. Within the range of the preliminary estimate, the time delay relationship of the signals is further used to compute a more precise DOA estimate. Figure 18a–d present the energy distribution and Sinc model fitting curves for four subordinate nodes in the anechoic tank experiment. The experimental results indicate that the system accurately estimated the azimuths of the four subordinate nodes, with values of 147.98°, 186.13°, 330.51°, and 283.56°, respectively. These estimates correspond precisely to the directions of beams 7.51, 9.21, 15.62, and 13.54 on the master node array. The measured mean absolute error was approximately 1.81°, with detailed statistics shown in Table 4.

Although the error was slightly higher than the numerical simulation results due to practical physical factors such as transducer channel inconsistencies and hardware installation biases, the system still maintained high positioning accuracy and successfully achieved sub-sector-level azimuth locking. The experimental results strongly validate the robustness of the proposed RSS-TDOA fusion localization algorithm in the real physical prototype, demonstrating the system’s capability to achieve precise spatial topology sensing in complex underwater acoustic environments.

**Figure 19:**
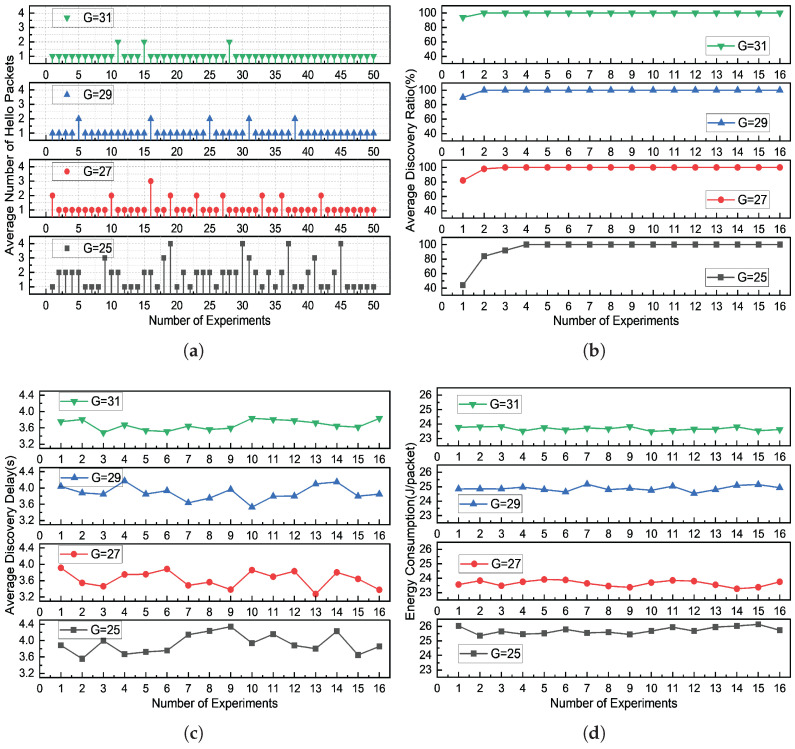
The average discovery delay and average energy consumption of neighbor nodes in the DBCB during the anechoic tank experiment. (**a**) Average number of Hello packets. (**b**) Average discovery ratio. (**c**) Average discovery delay. (**d**) Average energy consumption.

In the anechoic tank experiment, we set the detection threshold to 12 dB and conducted a comparative analysis of the discovery rate, average discovery delay, and energy consumption of the protocol under different transmission power levels to validate the efficiency of the DBCB node discovery protocol. As shown in Figure 19, with increasing transmission power, the average energy consumption of DBCB increases, the delay for node discovery decreases, and the overall discovery rate improves significantly. This indicates that at higher power levels, the signal coverage expands, the received signal-to-noise ratio (SNR) increases, and the probability of node discovery is enhanced. However, higher power is not always better. At different distances, transmitting high-power signals at short ranges can cause out-of-band leakage, leading to interference in other frequency bands during the processing. Therefore, designing a power-adaptive mechanism is essential to improve network performance.

Figure 19a,b show the average number of Hello packets sent and the average node discovery rate required to complete node discovery in each round of the experiment. As transmission power increases, the number of Hello packets required for node discovery gradually decreases. When G = 31 and G = 29, the neighbor node discovery task is completed within two cycles. When G = 27 and G = 25, the average neighbor node discovery rate within two cycles reaches 98%. Repeated trials indicate that within the verified power range, the DBCB protocol achieves a 100% node discovery rate within four cycles. Figure 19c presents the statistical results for the average discovery delay. Under low power conditions, some signals fail to reach the detection threshold of the primary node, leading to an increase in retransmissions and, consequently, a higher average discovery delay. When G = 27, the average discovery delay for network neighbor nodes is minimized, reducing the delay by 6.34%, 6.25%, and 1.04% compared to G = 25, G = 29, and G = 31, respectively. This highlights that high power can also lead to in-band interference, which should not be overlooked. Figure 19d illustrates the energy consumption of the protocol under different transmission power conditions. There is a significant positive correlation between transmission power and energy consumption. However, there is also a boundary to the benefits of power utilization. The DBCB protocol strikes a balance between maximum performance and energy consumption. By combining appropriate power configurations with multi-beam cooperative discovery mechanisms, the protocol significantly reduces control overhead and energy consumption while ensuring a high success rate. The anechoic tank experiment validates the effectiveness of the node discovery mechanism and the reasonableness of the power-adaptive strategy. This further demonstrates the efficiency and engineering feasibility of the protocol in multi-node network environments and provides important reference data for subsequent large-scale dynamic networking experiments.

## 5. Conclusions

This study proposes a Dynamic Beam Control-based Multi-Parallel Transceiver Neighbor Discovery Protocol (DBCB), which improves node discovery efficiency by dynamically adjusting transmission beams and optimizing spatiotemporal frequency resource allocation. Simulation results show that the performance of the DBCB protocol outperforms similar neighbor discovery protocols based on Directional Transmission-Receive (DTR) and Random Two-Way (RTW) mechanisms, with average discovery rate improvements of 7.84% and 28.17%, and average discovery delay reductions of 28.13% and 59.06%, respectively. The anechoic tank experiment demonstrates that multi-beam parallel transmission can effectively improve underwater node discovery efficiency, with simulation results aligning with experimental data, confirming the system’s stability and high efficiency. Although we have achieved significant performance improvements in simulations, hardware and computational resource limitations in real-world deployments may present challenges to the protocol’s execution efficiency and real-time performance. Future research will focus on optimizing the protocol for real-world system implementation to accommodate hardware computational capabilities and power consumption constraints. Moving forward, we will also deploy and validate the protocol in actual underwater environments and expand its application to complex network topologies. Additionally, we aim to enhance the protocol’s adaptability to underwater acoustic channel variations and environmental changes, further optimizing low power consumption and energy efficiency to improve the performance and reliability of underwater acoustic networks in real-world applications.

## Figures and Tables

**Table 1:** Detailed statistical table of received signals from different array elements.

NodeID	SAE	BID	POE (°)	SIA (°)	LAF	SAV (dB)
Node1	Left-3	5	90	60.00	0.1025	−19.78
	Left-2	6	112.5	37.50	0.2531	−11.93
	Left-1	7	135.0	15.00	0.8265	−1.66
	Center	8	157.5	−7.50	0.9540	−0.41
	Right-1	9	180.8	−30.00	0.4432	−7.07
	Right-2	10	202.5	−52.50	0.0229	−32.82
	Right 3	11	225.0	−75.00	0.1784	−14.97
Node2	Left-3	6	112.5	72.50	0.1713	−15.33
	Left-2	7	135.0	50.00	0.0119	−38.46
	Left-1	8	157.5	27.50	0.5104	−5.84
	Center	9	180.0	5.00	0.9793	−0.18
	Right-1	10	202.5	−17.50	0.7702	−2.27
	Right-2	11	225.0	−40.00	0.1962	−14.14
	Right 3	12	247.5	−62.50	0.1218	−18.29
Node3	Left-3	13	270.00	60.00	0.1025	−19.78
	Left-2	14	292.5	37.50	0.2531	−11.93
	Left-1	15	315.0	15.00	0.8265	−1.66
	Center	16	337.5	−7.50	0.9540	−0.41
	Right-1	1	0.0	−30.00	0.4432	−7.07
	Right-2	2	22.5	−52.50	0.0229	−32.82
	Right 3	3	45	−75.00	0.1784	−14.97
Node4	Left-3	10	202.5	77.50	0.1839	−14.71
	Left-2	11	225.0	55.00	0.0534	−25.45
	Left-1	12	247.5	32.50	0.3773	−8.47
	Center	13	270.0	10.00	0.9195	−0.73
	Right-1	14	292.5	−12.50	0.8766	−1.14
	Right-2	15	315.0	−35.00	0.3137	−10.07
	Right 3	16	337.5	−57.50	0.0798	−21.96

**Table 2:** Statistical table of node discovery rate and average discovery delay.

Method	Average Discovery Radio (ADR)			Average Discovery Delay (ADD)		
	M2	M4	M6	M2	M4	M6
DTR	0.87322	0.95892	0.92206	8.13076	6.79995	7.67872
RTW	0.72817	0.83577	0.58015	14.77995	10.88559	14.0241
DBCB (Ours)	0.99196	0.998	0.99938	5.57844	5.6126	5.05925

**Table 3:** Statistical table of the decrease in node discovery rate and the increase in average discovery delay as the number of nodes increases.

Method	Decrease Rate of Discovery Radio			Increment in Discovery Delay		
	M2	M4	M6	M2	M4	M6
DTR	0.34731	0.20502	0.29034	7.10839	4.92926	6.94779
RTW	0.70215	0.55752	0.6494	19.2857	10.79327	18.79518
DBCB (Ours)	0.05312	0.0125	0.00625	4.04298	3.86882	2.91612

**Table 4:** Detailed statistical table of beam matching information for detected signals by the master node in the anechoic tank experiment.

NodeID	Physical Val. (°)	Estimated Val. (°)	Matched Val. (No.)	Error (°)
Node1	150	147.98	7.51	2.02
Node2	185	186.13	9.21	1.13
Node3	330	330.51	15.62	0.51
Node4	280	283.56	13.54	3.56

## Data Availability

The data presented in this study are available from the corresponding author upon reasonable request.
